# Ethanol and acetaldehyde mediate folic acid and human papillomavirus-induced proliferation of oral squamous cell carcinoma cells *in vitro*

**DOI:** 10.1186/1753-6561-6-S3-P64

**Published:** 2012-06-01

**Authors:** Karl Kingsley, Michael Struthers, Nicholas Freel, John Enyeart, William Munford, Cassie N Miller, Bryan Spangelo, Joshua Steffen, Griffin Park, Mark A Keiserman, Christine J Bergman

**Affiliations:** 1 Department of Biomedical Sciences, School of Dental Medicine, University of Nevada, Las Vegas, USA; 2 School of Life Sciences, College of Sciences, University of Nevada, Las Vegas, USA; 3 Columbia University, College of Dental Medicine, New York, USA; 4 Department of Biology, College of Sciences, University of Nevada, Reno, USA; 5 School of Health Related Professions: Nutrition, University of Medicine and Dentistry, New Jersey, USA; 6 Department of Food and Beverage, Harrah Hotel College, University of Nevada, Las Vegas, USA

## Background

Although great scientific emphasis has been placed upon HPVas the primary cause of cervical cancers and its involvement in carcinogenic progression of other cancers, less attention has been focused on the secondary factors that are associated with progression from subclinical HPV infection to invasive carcinoma. Among the secondary factors that limit virus production and carcinogenic progression is CpG methylation of the HPV genome. Several studies now confirm that CpG site-specific methylation of HPV DNA, mediated in part by folate availability, is sufficient to suppress neoplastic progression. In contrast, demethylation or hypomethylation of HPV-DNA sequences is required for transformation, revealing the importance of preferential DNA methylation at CpG sites in the HPV long control region (LCR) between L1 and E6 HPV genes, in addition to the tumor suppressor sites in p53 exons 248 and 273. Because HPV has the potential to initiate oncogenesis, and also to modulate oral cancer growth and folate plays a central role in mediating the availability of methyl groups for CpG-specific DNA methylation (modulating both p53 and HPV mRNA expression) – an investigation of these inter-connected and inter-related mechanisms in oral cancers must be undertaken. In addition, ethanol and acetaldehyde may also play critical roles in determining folate availability, and are primary risk factors for the development of oral cancers, which makes the evaluation of these interconnected metabolic pathways critically important.

## Materials and methods

Using a comprehensive series of integrated *in vitro* assays, including proliferation, viability and mRNA analysis using RT-PCR, distinct effects of ethanol and acetaldehyde administration were observed in the oral cancer cell lines, CAL27, SCC15 and SCC25. In addition, the growth modulating effects of HPV infection and FA supplementation were also examined.

## Results

Both high-risk HPV strains 16 and 18 induced robust growth-stimulating effects in CAL27 cells, while strain-specific responses were observed in SCC25 and SCC15 cells. FA administration [0-400 µg/mL] significantly increased the growth rate of all cell lines evaluated. In addition, FA administration induced broad, general increases in cell viability among all cell lines that were associated with *p53* mRNA transcriptional down-regulation. None of these cell lines were found to harbor the common C677T mutation in methylenetetrahydrofolate reductase (*MTHFR*), which can reduce FA availability and may increase oral cancer risk.

Administration of ethanol or acetaldehyde [10 – 100 µM] significantly inhibited oral cancer cell growth in all cell lines tested. Moreover, the growth inhibiting properties of ethanol and acetaldehyde also mediated and reduced the HPV- and FA-induced growth of CAL27, SCC15 and SCC25 cells. These changes were associated with a transcriptional up-regulation of alcohol dehydrogenase (ADH) and other enzymes involved in alcohol metabolism.

## Conclusions

Although tobacco and alcohol use are the main risk factors for developing oral cancer, HPV infection and FA availability may modulate their growth and progression. This study provides preliminary evidence that alcohol and acetaldehyde may mediate and down-regulate the growth enhancing effects of either HPV or FA *in vitro* and may be associated with up-regulation of metabolic pathways involved with alcohol metabolism.

